# Nonoperative Management Strategies for Anastomotic Leaks After One Anastomosis Gastric Bypass (OAGB): A Literature Review

**DOI:** 10.7759/cureus.69708

**Published:** 2024-09-19

**Authors:** Amir Farah, Kamil Malshy, Anna Tatakis, Wisam Abboud, Amir Mari, Sa'd Sayida

**Affiliations:** 1 General Surgery, Medical College of Wisconsin, Milwaukee, USA; 2 Urology, University of Rochester Medical Center, Rochester, USA; 3 General Surgery, Edinburgh Medical Missionary Society (EMMS) Nazareth Hospital, Nazareth, ISR; 4 Gastroenterology and Hepatology, Edinburgh Medical Missionary Society (EMMS) Nazareth Hospital, Nazareth, ISR

**Keywords:** bariatric, bariatric complication, endoscopy, fistula, leak, mini gastric bypass, nonoperative management bariatric, obesity, one anastomosis gastric bypass, surgery

## Abstract

One Anastomosis Gastric Bypass (OAGB) has gained widespread acceptance as an effective bariatric surgery due to its relative simplicity and favorable outcomes in weight loss and metabolic improvement. However, anastomotic leaks, though uncommon, present a significant complication with the potential for severe morbidity and mortality if not managed appropriately. This review examines the range of nonoperative strategies currently employed to manage anastomotic leaks and fistulae following OAGB. The focus is on endoscopic techniques, including the use of clips, stents, suturing systems, internal drainage, vacuum therapy, and tissue sealants, which have been successfully used in various gastrointestinal surgeries. Although a proportion of patients will require surgical treatments, these strategies offer less invasive alternatives to surgical intervention and can be tailored to the specific characteristics of the leak and patient condition. However, the application of these techniques specifically for OAGB-related leaks is not as well-documented. This review lists the available evidence on these nonoperative approaches, highlighting some of their potential benefits and limitations. While these methods show promise, there is a clear need for further research to establish standardized protocols and optimize their use in the context of OAGB-related leaks and fistulae.

## Introduction and background

The rates of obesity are on the rise worldwide, and obesity-related comorbidities (cardiovascular disease (CVD), type 2 diabetes mellitus (T2DM), and cancer) constitute the leading causes of death across the globe [1]. According to the World Health Organization (WHO), 890 million adults (16% of the world’s population) are classified as obese, with a Body Mass Index (BMI) of 30 kg/m² or higher [2]. It has been shown that bariatric surgery is associated with decreased CVD events, reduced deaths due to certain cancers, and T2DM, in addition to decreased all-cause mortality [3].

Since the inception of the original gastric bypass by Mason and Ito in 1967 [4], several other procedures have emerged from that time. The One Anastomosis Gastric Bypass (OAGB) has become a relatively popular bariatric procedure worldwide and is now being performed routinely in most countries around the world [5]. Historically, it was first performed in the United States (US) by Rutledge in 1997 [6]. With some variations across the years, the surgical technique involves creating a long gastric pouch starting below the crow’s foot and extending up to the angle of His [5]. A wide gastro-jejunal anastomosis is then formed, connecting the pouch to an anti-colic loop of jejunum, positioned 150-200 cm distal to the ligament of Treitz [5]. The procedure offers several advantages over its counterpart, the Roux-en-Y Gastric Bypass (RYGB) [7]. These include a faster learning curve, reduced technical difficulty, and ease of conversion, with non-inferior weight loss outcomes [8]. In fact, in one meta-analysis comparing RYGB to OAGB, it was found that patients undergoing OAGB had a higher 1 and 2-year Excess Weight Loss % (EWL%), higher type 2 diabetes mellitus remission rate, as well as a shorter operation time [9]. There was no difference, however, in hypertension remission, mortality, leak rate, or hospital stay between the two procedures [9]. Nevertheless, OAGB has created controversy within the bariatric surgery community due to concerns about bile reflux, marginal ulcers, and the subsequent risk of gastric and esophageal cancers [7].

Leaks after OAGB most commonly occur at the Gastro-Jejunal Anastomosis (GJA), and as opposed to leaks after RYGB, they are high-flow, bile-containing leaks, originating from the contents of the afferent limb [10]. These bile-rich leaks can lead to rapid tissue destruction and sepsis, making early intervention critical [10]. These leak characteristics may also further complicate certain endoscopic solutions such as clips or sutures, as they are less effective in high-flow environments [10].

Leaks increase overall morbidity of bariatric surgeries to 61% and mortality to 15% [11]. The occurrence of an Anastomotic Leak After OAGB (ALAOAGB) is relatively rare (<1%) [12]. A randomized study by Lee et al. (2005) showed a lower operative morbidity rate with OAGB compared to RYGB (7.5% vs 20%) and also concluded that OAGB is non-inferior to RYGB during its follow-up (two years) [8]. Still, ALAOAGB is regarded as the procedure’s most feared complication [12].

A lack of a consensus in the literature makes the exact definition of an early versus late leak quite difficult, but regarding their pathogenesis, early anastomotic leaks are usually due to technical factors, and late leaks are most often due to impaired healing [13]. As with most anastomotic leaks throughout the gastrointestinal tract, operative treatment remains the treatment of choice for acute uncontained anastomotic leaks (early or late), especially in unstable patients [14]. In high-risk stable patients, on the other hand, reoperation could significantly increase the likelihood of acquiring additional complications, and may lead to increased mortality [10-12]. In this specific patient population, nonoperative management could prove to be potentially lifesaving [10-12]. Nonoperative techniques provide an alternative to surgery in several situations, reducing the physical stress on patients while offering effective treatment options [10-12].

The purpose of this review is to present the nonoperative treatment approaches for ALAOAGB, focusing on those interventions performed for stable patients without diffuse peritonitis. Furthermore, it will demonstrate the gaps in the literature regarding the utility of these treatments for ALAOAGB specifically.

## Review

Intraoperative maneuvers

Efforts by bariatric surgeons to intraoperatively detect leaks are debatable, and their effectiveness has been challenged in the current literature [15-17]. An intraoperative leak test (IOLT) may be performed intraoperatively under endoscopic guidance, or by orogastric tube placement with either air insufflation or by using a colored dye. Its routine use for sleeve gastrectomy (SG) has been studied and yielded a sensitivity and positive predictive value of 0% in some series [18]. IOLT use for RYGB is supported by literature, with leak detection rates between 5.9% and 15%, which also correspond to evidence supporting its use in colorectal anastomoses [19-21]. A recent retrospective study using a large cohort of patients in which IOLT was done routinely found that IOLT was, in fact, associated with increased rates of postoperative leak for SG and RYGB (odds ratio (OR)=1.48 and 1.9), lower rates of bleeding for SG (OR 0.76), and the association between leak testing and reoperation or readmission was not significant [22]. Surgical drains are used more selectively, and their use was also associated with a higher leak rate, and a postoperative swallow study had no impact on the leak rate [22]. While its large sample size is one of its strengths, its observational retrospective methodology may be prone to selection bias and possible confounding, such as patient comorbidities and surgical techniques. The lack of randomization also limits the ability to attribute higher leak rates directly to IOLT. Future studies are needed to evaluate these associations. These methods have not been studied in OAGB specifically.

Treatment overview

Endoscopic treatments have been studied and used successfully after various upper gastrointestinal (UGI) surgical complications and are considered first line in many circumstances [23,24]. Cereatti et al. demonstrated the effectiveness of endoscopic management in treating leaks and fistulae after SG and RYGB, with reported success rates of up to 85% [23]. Nedelcu et al. highlighted the success of endoscopic treatments in managing chronic leaks following SG, with an emphasis on the durability of these interventions where surgical re-intervention was avoided [24]. Similarly, Kirschniak et al. detailed the use of endoscopic clips in full-thickness closure of gastrointestinal defects after gastric surgeries, showing success rates close to 90% in acute leak cases [25]. These studies highlight some of the success of endoscopic techniques, but although they may be suggestive of their use in OAGB, the literature regarding their specific use for ALAOAGB is scarce. Further studies, especially long-term comparative studies, are needed to evaluate their role for ALAOAGB. Nevertheless, endoscopic interventions play several roles in managing postoperative leaks and fistulae after UGI surgery.

The main endoscopic treatments available for the management of UGI leaks and fistulae are endoscopic clips, stents, suturing systems, endoscopic internal drainage, endoscopic vacuum systems, and tissue sealants [23].

Clips

The main types of endoscopic clips are through-the-scope (TTS) clips, and over-the-scope (OTS) clips. The main advantages of OTS clips over TTS clips are that OTS clips have wider arms and they accomplish a more durable full-thickness closure than TTS clips [25]. However, the removal of an OTS clip is challenging, and has a high rate of fistula recurrence [26]. They may also interfere with future surgical procedures [23]. TTS clips are easy to use and may be used in a variety of clinical situations, but their application is limited in chronic leaks due to their limited grasping pressure when applied on unhealthy or inflamed tissue [23]. Nevertheless, they have a high reported success rate, approaching 95% in managing leaks after gastric surgery [27]. OTS clips have more powerful full-thickness grasping strength and may be used in defects up to 2 cm in size [23]. Their use depends on whether they are used as a primary treatment, and if they are used in an acute or chronic setting [28]. In the acute setting (within three hours of surgery), success rates approach 100%, while in the chronic setting, a significantly lower success rate was observed [29]. The utility of these techniques has yet to be studied in ALAOAGB.

Stents

Luminal stents such as self-expandable plastic stents (SEPS) and self-expandable metal stents (SEMS) have been used throughout the upper and lower gastrointestinal tract as a solution for leaks and fistulae, as well as for a variety of clinical scenarios [30,31]. SEPS are composed of a polyester net that is fully covered with silicone, and SEMS are composed of Elgiloy® or Nitinol [23]. SEPS can be more easily removed, are cheaper, and less frequently induce tissue hyperplasia [23]. These benefits can be overshadowed by their disadvantages, which include their larger diameter, limited use in strictures, and high rates of migration, reaching 40% [31]. As opposed to SEPS, reports of SEMS-induced tissue hyperplasia with ingrowth can be as high as 41-53% [32,33], and despite evolving treatment options for this condition, it is still associated with numerous complications upon stent removal [34]. For bariatric surgery, special SEMS have been developed with the goal of lower migration rates, although similar rates of migration have been reported despite their use [35,36]. The utility of these techniques has yet to be studied in ALAOAGB.

Sutures 

Endoscopic suturing has evolved in recent years; it requires a high level of expertise and training, and its use is still limited with regard to leak and fistula management [37,38]. The cornerstone of endoscopic suturing is using viable and healthy tissue for closure; hence, de-epithelization of the epithelized fistula is often necessary [39]. Success rates vary throughout the literature, and more studies are needed to validate this technique’s role in the treatment of leaks and fistulae throughout the gastrointestinal tract. In a large multicenter retrospective study by Sharaiha et al. of patients who underwent endoscopic suturing for the management of luminal defects and/or stent anchoring, clinical success with endoscopic suture use was noted to be 93% in perforations, 80% in fistulas, and only 27% in anastomotic leak closure [38]. Studies regarding the impact of this method specifically on ALAOAGB have yet to be undertaken.

Internal Drainage Systems (IDS) 

Employing pigtail stents across a luminal defect internally drains fluid collections into the lumen; it also acts as a barrier that seals the leak orifice, facilitating earlier alimentation, promoting re-epithelialization of the tract, and finally its closure [40,41]. Several studies have validated the use of IDS for peri-gastric fluid collections after bariatric surgery, some achieving significantly greater efficacy than with the use of SEMS, sealants, and OTS clips [41-43]. In a study by Donatelli et al., clinical success (defined as the absence of free contrast medium extravasation in the peritoneal cavity) was achieved in 78.2% of patients with leaks after SG, although some required multiple treatments [41]. The role of IDS in ALAOAGB specifically has yet to be studied.

Endoscopic Vacuum Therapy (EVT) 

Historically used mainly for esophageal and distal colorectal leaks, this minimally invasive, endoscopic technique works by exerting constant negative pressure within a lumen, providing continuous drainage, promoting re-epithelialization by inducing angiogenesis, edema reduction, and subsequent second intention closure of the luminal defect [23,44]. There are two types of systems: the vacuum sponge and the vacuum stent, with two approaches, the intracavitary approach and the intraluminal approach, with no superiority demonstrated between the methods [44]. Regarding its use in bariatric surgery, studies report success rates approaching 90-100% using EVT for leaks after bariatric surgery, but with less specific emphasis on ALAOAGB [45,46]. More research is needed in this specific area of EVT use.

Tissue Sealants (TS) 

There are two main types of TS: biological glue (fibrin) and synthetic glue (cyanoacrylate) [23]. The main advantage of fibrin is that it works by physiologically imitating wound healing, without subsequent inflammation [47]. Cyanoacrylate, on the other hand, can induce tissue necrosis and inflammation but has the advantage of stronger adhesive and antibacterial properties, as well as the capability of working in a wet environment [48]. Success rates with their use differ in the literature, with some reporting success rates up to 75%, while others report durable closure in only 36.5% when used as a single therapy [49,50].

Table 1 lists the advantages and disadvantages of the strategies described for leak and fistula management [23-50].

**Table 1:** Strategies in leak and fistula management References [23-50]. TTS: Through The Scope, OTS: Over The Scope, ALAOAGB: Anastomotic Leak After One Anastomosis Gastric Bypass, OAGB: One Anastomosis Gastric Bypass, SEM: Self Expandable Metal Stent, SEPS: Self Expandable Plastic Stent, SG: Sleeve Gastrectomy.

Strategy	Description	Advantages	Disadvantages	ALAOAGB
Intraoperative Leak Testing	Endoscopic or orogastric tube placement with air insufflation or colored dye to detect leaks intraoperatively	Supported for RYGB; may detect leaks early	Sensitivity and predictive value may be low; associated with increased postoperative leaks in some studies	Not specifically studied in OAGB
Endoscopic Clips	TTS and OTS clips used to close leaks	High success rates (up to 95%) in gastric surgery; OTS clips offer full-thickness closure	OTS clips difficult to remove; risk of fistula recurrence; limited use in chronic leaks	Utility in ALAOAGB not fully studied
Stents	SEPS and SEMS used to seal leaks and support fistula closure	SEPS easy to remove; less tissue hyperplasia; SEMS developed to reduce migration	High migration rates with SEPS; tissue hyperplasia with SEMS; complications with stent removal	Techniques not specifically studied in ALAOAGB
Endoscopic Suturing	Suturing leaks and fistulae using viable tissue; often requires de-epithelization	High success rates in managing perforations and fistulae	Requires expertise; lower success rates in anastomotic leaks (27%)	More research is needed to validate in ALAOAGB
Internal Drainage Systems	Pigtail stents across defects to internally drain fluid collections into the lumen promoting re-epithelization and closure	Higher efficacy compared to SEMS, sealants, and OTS clips; promotes earlier alimentation	May require multiple treatments; primarily studied in SG	Specific role in ALAOAGB yet to be studied
Endoscopic Vacuum Therapy	Negative pressure therapy within the lumen to promote re-epithelialization and closure	High success rates (90-100%) in leaks after bariatric surgery	Limited to experienced centers	Limited emphasis on ALAOAGB; more research needed
Tissue Sealants	Fibrin and cyanoacrylate glues used to seal leaks by promoting or imitating wound healing	Fibrin promotes physiological healing; cyanoacrylate is a strong adhesive with antibacterial properties	Variable success rates (36.5 to 75%); cyanoacrylate may cause tissue necrosis and inflammation	Success rates and efficacy need further studies in ALAOAGB

## Conclusions

As global obesity rates rise, bariatric surgeries have become a key intervention for sustained weight loss and the reduction of obesity-related comorbidities. Anastomotic leaks and fistulae are among the most serious complications. Managing these complications requires a multidisciplinary approach, favoring minimally invasive techniques that align with the principles of bariatric surgery.

While various treatment modalities exist for managing anastomotic leaks, tailored approaches based on the patient's condition are essential. Despite extensive literature on leaks following procedures like RYGB and SG, there is a notable gap in research specifically addressing leaks after OAGB. Further investigation is needed to identify optimal management strategies, and exploring novel treatments like stem cell therapy could enhance healing and patient outcomes.
